# Metabolomic Variation Aligns with Two Geographically Distinct Subpopulations of *Brachypodium Distachyon* before and after Drought Stress

**DOI:** 10.3390/cells10030683

**Published:** 2021-03-19

**Authors:** Aleksandra Skalska, Manfred Beckmann, Fiona Corke, Gulsemin Savas Tuna, Metin Tuna, John H. Doonan, Robert Hasterok, Luis A. J. Mur

**Affiliations:** 1 Plant Cytogenetics and Molecular Biology Group, Faculty of Natural Sciences, Institute of Biology, Biotechnology and Environmental Protection, University of Silesia in Katowice, 40-032 Katowice, Poland; askalska@us.edu.pl; 2 Institute of Biological, Environmental and Rural Sciences (IBERS), Aberystwyth University, Aberystwyth SY23 3DA, UK; meb@aber.ac.uk; 3 National Plant Phenomics Centre, Institute of Biological, Environmental and Rural Sciences (IBERS), Aberystwyth University, Aberystwyth SY23 3EB, UK; fic5@aber.ac.uk (F.C.); jhd2@aber.ac.uk (J.H.D.); 4 Tekirdag Anatolian High School, 59030 Suleymanpasa, Tekirdag, Turkey; glsvs@yahoo.com; 5 Department of Field Crops, Faculty of Agriculture, Tekirdag Namik Kemal University, 59030 Suleymanpasa, Tekirdag, Turkey; mtuna@nku.edu.tr; 6 College of Agronomy, Shanxi Agricultural University, Taigu, Jinzhong 030801, Shanxi, China

**Keywords:** *Brachypodium distachyon*, metabolome, metabolotypes, drought, osmolytes, amino acids, auxin

## Abstract

*Brachypodium distachyon* (Brachypodium) is a non-domesticated model grass that has been used to assess population level genomic variation. We have previously established a collection of 55 Brachypodium accessions that were sampled to reflect five different climatic regions of Turkey; designated 1a, 1c, 2, 3 and 4. Genomic and methylomic variation differentiated the collection into two subpopulations designated as coastal and central (respectively from regions 1a, 1c and the other from 2, 3 and 4) which were linked to environmental variables such as relative precipitation. Here, we assessed how far genomic variation would be reflected in the metabolomes and if this could be linked to an adaptive trait. Metabolites were extracted from eight-week-old seedlings from each accession and assessed using flow infusion high-resolution mass spectrometry (FIE-HRMS). Principal Component Analysis (PCA) of the derived metabolomes differentiated between samples from coastal and central subpopulations. The major sources of variation between seedling from the coastal and central subpopulations were identified. The central subpopulation was typified by significant increases in alanine, aspartate and glutamate metabolism and the tricarboxylic acid (TCA) cycle. Coastal subpopulation exhibited elevated levels of the auxin, indolacetic acid and rhamnose. The metabolomes of the seedling were also determined following the imposition of drought stress for seven days. The central subpopulation exhibited a metabolomic shift in response to drought, but no significant changes were seen in the coastal one. The drought responses in the central subpopulation were typified by changes in amino acids, increasing the glutamine that could be functioning as a stress signal. There were also changes in sugars that were likely to be an osmotic counter to drought, and changes in bioenergetic metabolism. These data indicate that genomic variation in our Turkish Brachypodium collection is largely reflected as distinctive metabolomes (“metabolotypes”) through which drought tolerance might be mediated.

## 1. Introduction

*Brachypodium distachyon* (hereafter Brachypodium) is a well-established model for grasses. It has a small stature with a relatively large seed and spikelet and has undemanding growth requirements that lends itself to research in many laboratories [1,2]. After years of development by the international community, Brachypodium is represented by a large repository of well-annotated genome sequences and well-established plant transformation and gene-editing protocols [3,4,5].

As a non-domesticated species, Brachypodium retains its importance in assessing how environmental factors impacts genomic variation. Thus, an initial resequencing project based on 54 inbred Brachypodium accessions established variation linked to series of populations. Within Turkey, variation was linked to a Turkish (T+) population and an extremely delayed flowering (EDF+) phenotype [4]. This split was also detected in a Genotyping By Sequencing (GBS) analysis of >1400 accessions where a split was observed in Western and Eastern Mediterranean populations but within each population there existed the same A and B subgroup [5]. Crucially, these were not linked to any environmental or climatic variables. In a recent study, we have established a new collection of Turkish accessions where we sampled to reflect different climatic regions [6]. Genome sequencing established that variation was again associated with two subpopulations and these were grouped into geographic regions, as either coastal or central. Further, bioclimatic variables, “bioclim”, assessments correlated these subpopulations with either the temperature of the coldest month (coastal subpopulation) where EDF+ genotypes predominated or precipitation in the driest month (central) where T+ genotypes were most common. In response to suggestions that methylome variation could be linked to the environment difference [7,8], we also investigated methylomic differences in our Turkish population. This supported the idea that genetic and methylomic variation was indeed reflecting the coastal/central split in subpopulations [6].

A key question is how variation acts to potentially influence phenotypic variation linked to environment. Adaptions to the environment are often described at the level of the genome or transcriptome [9,10] but these often act though biochemical changes [11]. Focusing only on responses to stress, biochemical changes are important to preserve osmotic homeostasis, membrane integrity or countering the oxidative stress [11,12]. Considering metabolite changes, studies have indicated the importance of sugars, sugar alcohols (particularly mannitol) and amides such as proline and polyamines. These metabolites act as osmolytes to reduce cellular dehydration, compatible solutes which stabilise enzymes, membranes and other cellular components as well as chelating agents [13]. Other metabolites, such as ascorbate and glutathione, have important anti-oxidant roles [14].

Metabolomics offers a means to comprehensively assess the biochemical variation between samples from different germplasm, organs or treatments. Metabolomics represents the summation of genomic, transcriptional and proteomic changes and can therefore offer profound insights into underlying mechanisms governing plant phenotypes [15,16]. Metabolomics is based on the detection and relative quantification of metabolites in a complex biosamples using mass spectroscopy (MS) or nuclear magnetic resonance (NMR) based approaches. Prior to MS, samples can be fractionated by gas chromatography or liquid chromatography but in terms of dynamic range and resolution, flow-infusion-high resolution MS (FIE-HRMS) has particular advantage when attempting to derive a wide ranging snap-shot of metabolism. This is important as plants are particularly rich and diverse in their metabolome with estimates of the total number of metabolites found in plants suggesting as many a ~200,000 [17,18]. Focusing only on plant stress, metabolomics has been used in a range of scenarios, including assessing the responses to drought (e.g., [19,20,21]), chilling (e.g., [22,23]) or salt (e.g., [24,25]). All these stresses could influence the distribution patterns of particular plant populations.

Within the context of Brachypodium, metabolomics has been used to distinguish between the three species of *Brachypodium* genus [26], characterise drought-induced senescence [27] and, in a comparative metabolomic approach, to show common responses to infection with the rice blast pathogen *Magnaporthe grisea* [28]. Our group has previously used metabolomics for in depth assessments of the responses of Brachypodium to *M. grisea* [29,30] and also to link metabolomes to phenomic and transcriptional changes occurring following the imposition of drought [31,32]. Our recent paper focused on genomic and methylome variation in Turkish subpopulations [6] but our expertise in metabolomics offered the possibility of metabolotyping Brachypodium accessions. Metabolotyping (also known as metabotyping in human and veterinary research [33]) aims to link particular genetic or transcriptional patterns to specific metabolic types [34]. Thus, we sought to test if our genomic/methylome subpopulations would be reflected as particular metabolotypes that could be contributing to the adaptation to a particular environment.

Using FIE-HRMS, we profiled the metabolomes of seedling leaves from our Turkish collection of Brachypodium grown under identical environmental conditions. The metabolomes of the Brachypodium accession could be separated into those which were from coastal or central subpopulations. The more drought tolerant central subpopulation exhibited changes that could confer an osmo-protective role, bioenergtic capacities and also auxin levels which could improve root development. Thus, the Turkish Brachypodium population is represented by two metabolotypes that, in the case of the central subpopulation, could be playing a role in environmental variation.

## 2. Material and Methods

### 2.1. The Turkish Accessions

The origins of the 55 Turkish Brachypodium accessions are described in [6]. Three seeds from each accession (T_0_ generation sampled directly from Turkey) were germinated under controlled environmental conditions (Levingtons F2 with horticultural grit [1/5 vol] added prior to use, 16 h photoperiod, natural light supplemented with artificial light from 400-W sodium lamps at 22 °C). Seeds from this were collected to yield the T_1_ generation.

### 2.2. Metabolomic Analyses

Two large metabolomic experiments are described in this paper. In the non-stressed plants, the leaves from eight-week-old-seedlings from the T_0_ generation were collected from each accession. For the second, the drought experiment, the leaves from eight-week-old seedlings from the T_1_ generation exposed to drought stress for seven additional days after which they were sampled (see Section 2.3). Metabolites from frozen and ground leaves were extracted using a single-phase extraction solution (chloroform/methanol/water, 1:2.5:1, *v*/*v*/*v*). Frozen leaves were homogenized and mixed with 1 mL of the extraction solution for 20 min at 4 °C. Further, the samples were centrifuged for 30 min at 4 °C and the supernatant was transferred into new tubes from which 200 µL were taken for further analysis. Metabolite fingerprinting was performed by FIE-HRMS using a Q Exactive Plus Hybrid Quadrupole Orbitrap Mass Analyser with an Acella UHPLC system (Thermo Fisher Scientific, Bremen, Germany). The *m*/*z* (mass-ion) values were generated in both positive and negative ionization modes as was described by Baptista et al. [35]. The derived data are provided in Supplementary Materials Table S1. Individual metabolite *m/z* values were normalised as a percentage of the total ion count for each sample. Data were normalised to total ion count and log_10_-transformed. Multivariate analysis was performed using MetaboAnalyst 4.0 (http://www.metaboanalyst.ca/, accessed on 3 March 2021). The significance of the cross-validated *p*-values, based on the one-way analysis of variance (ANOVA) was set to *p* < 0.05. The multiple comparison and post hoc analysis used Tukey’s Honestly Significant Difference (Tukey’s HSD). Identification was based on the MS peaks to pathway algorithm [36] (tolerance = 5 ppm, reference library; *Oryza sativa*). This involved metabolites being annotated using KEGG database (https://www.genome.jp/kegg/pathway.html, accessed on 3 March 2021), considering the following possible adducts: [M+]+, [M+H]+, [M+NH4]+, [M+Na]+, [M+K]+, [M-NH_2_+H]+, [M-CO_2_H+H]+, [M-H_2_O+H]+; [M−]−, [M−H]−, [M+Na−2H]−, [M+Cl]−, [M+K−2H]−. Correlations between multiple adducts of a metabolite were used in the identification process.

### 2.3. Drought Experiment

The experimental set up for the drought experiment was also described in [6]. Briefly, 12 replicates of each accession from the T_1_ generation were germinated in pots with 50 g of 4:1 Levington F2: grit sand. After two weeks, seedlings were vernalised for a further six weeks, and then the eight-week-old plants were transferred into plant screening system at the National Plant Phenomics Centre (NPPC), Aberystwyth, UK. The NPPC allows computer regulated watering of each seedling pot. Watering was withdrawn from four replicates from each genotype to achieve 15%, 40% or 75% soil water contents (SWC) by seven days. This level of SWC was maintained until the end of the experiment at 12 days.

## 3. Results

### 3.1. Metabolite Profiling of Brachypodium Distachyon

**Figure 1:**
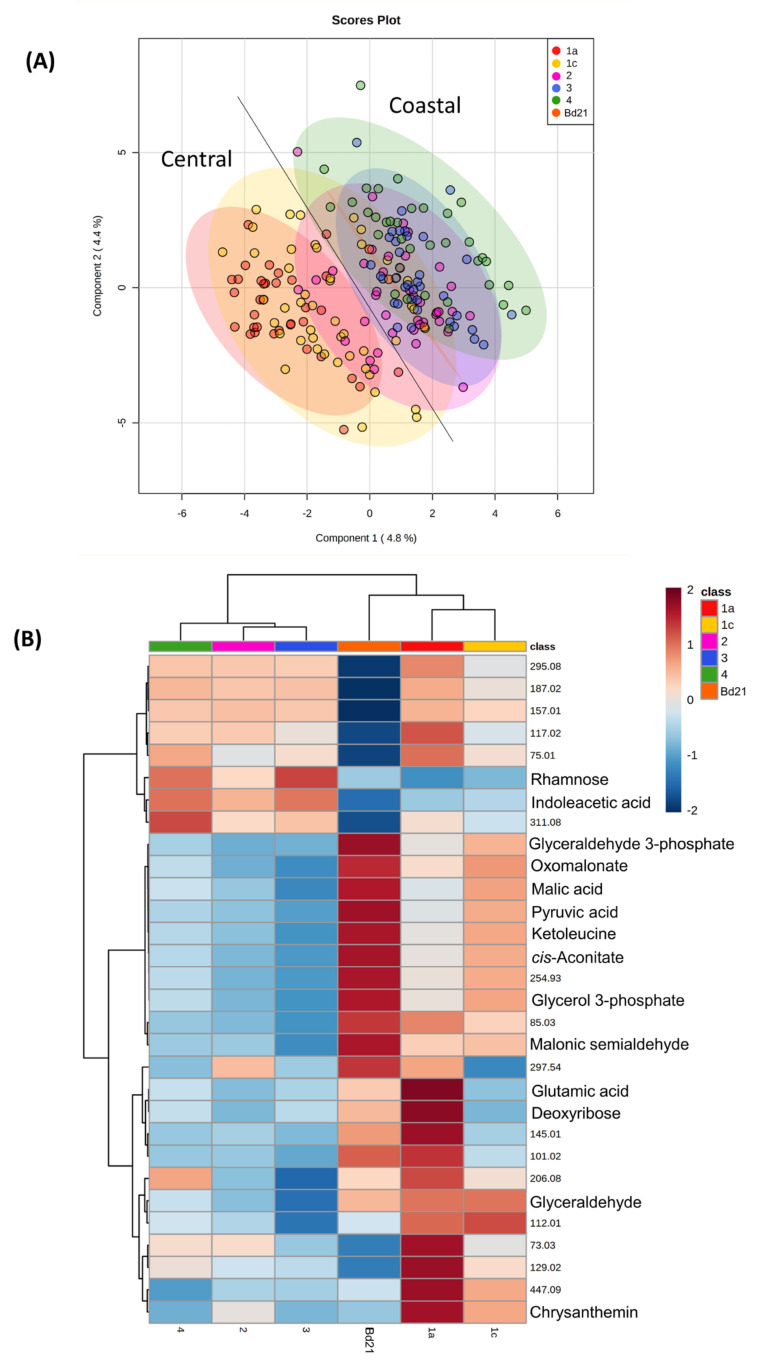
Metabolomic variation in Brachypodium accessions from different climatic regions of Turkey. Metabolite profiles from the leaves of eight-week-old Brachypodium seedlings were derived by Flow Infusion Electrospray-High Resolution Mass Spectroscopy (FIE-HRMS). Accessions are classified according of Turkish climate region of origin; 1a (red), 1c (yellow), 2 (purple), 3 (blue) and 4 (green) [6]. Profiles from the reference accession Bd21 are represented by dark orange points or block as relevant. Profiles were initially assessed using (**A**) Partial Least Square—Discriminant Analysis (PLS-DA). The major sources of variation were identified using one-way ANOVA correcting for false discovery rates (FDR). **(B)** Key variables are displayed as a heatmap. Some mass-ions (*m/z*) were identified from comparison with databases and are labelled with names. Where *m/z* could not be identified these are given as the raw data values.

Metabolite analyses of 55 Brachypodium accessions derived from natural Turkish environmental conditions were performed on fourth leaf samples collected eight weeks after germination. In our analysis, we also included the canonical Iraqi line—Bd21 which was the first accession to be sequenced. All seedlings were grown at an identical time and under identical environmentally controlled conditions. Metabolite profiles were derived following FIE-HRMS and assessed by Partial Least Squares—Discriminant Analysis (PLS-DA) (Figure 1A). The samples data points based on the accession’s Turkish region of origin, were designated as 1a, 1c, 2, 3 and 4 as described previously [6]. It was observed that region 1a was the most distinctive but there was considerable overlap with samples from region 1c. Samples from regions 2, 3 and 4 showed considerable overlap but there were some metabolomic similarities with region 1c. These observed divisions corresponded to the previously proposed separation into two subpopulations: central (consisting of regions 1a, 1c and the reference Bd21), and coastal (consisting of regions 2, 3 and 4) [6].

The sources of variation between the regions and subpopulations were targeted by ANOVA (*p* < 0.05) and some *m/z* were identified based on database comparisons of the parental ion masses and correlations with predicted ionisation patterns. These major sources of variation are displayed using a heatmap (Figure 1B). The clustering patterns provided clearer evidence of metabolomic separation of the Turkish accessions into coastal (2, 3 and 4) and central (1a, 1c) subpopulations, with Bd21 more closely aligned to the central but having some distinctive characteristics. Examination of the identified metabolites suggested that components of the tricarboxylic acid (TCA) cycle (citrate, malic acid, pyruvate, 2-oxoglutarate, *cis-*aconitate) were more abundant in the central compared to the coastal accessions.

**Figure 2:**
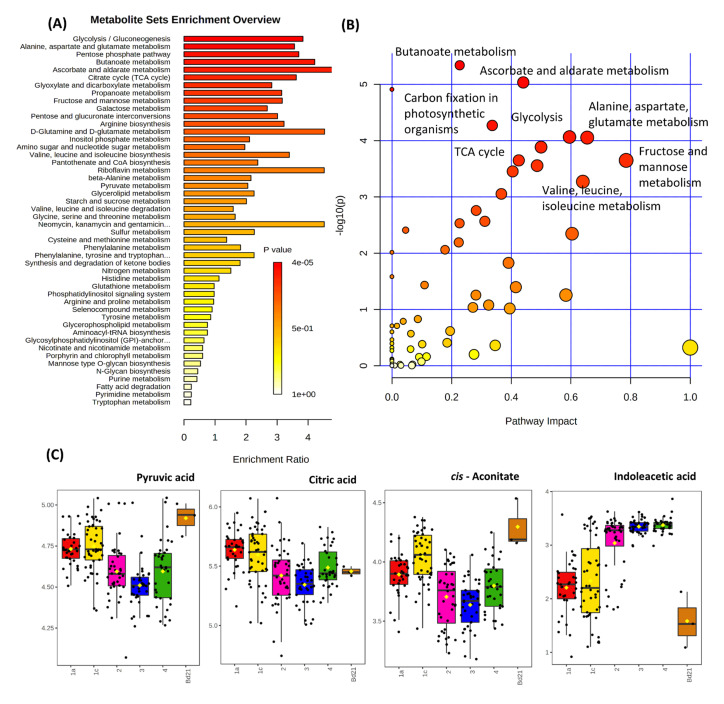
Biochemical pathway and metabolite analyses showing discrimination between Brachypodium metabolomes from different climatic regions of Turkey. FIE-HRMS derived metabolites which exhibited significant (one-way ANOVA correcting for false discovery rates, FDR) differences between accessions from different Turkish regions [6] were assessed using (**A**) metabolite set enrichment analysis (MSEA) or (**B**) the mummichog algorithm where significantly enriched pathways are also ranged for biological impact. (**C**) Exemplar metabolites exhibiting significant differences between accessions from different regions are shown using box and whisker plots. Regions 1a (red), 1c (yellow), 2 (purple), 3 (blue) and 4 (green) are indicated with Bd21 (dark orange) as the reference accession.

To provide wider functional information, pathway enrichment (Figure 2A) and pathway impact analyses (Figure 2B) were undertaken based on the significant metabolites. The most robust statistical valid results (Holm *p* and false discovery rates (FDR) (both < 0.05)) were seen for glycolysis/gluconeogenesis, alanine, aspartate and glutamate metabolism, pentose phosphate pathway and butanoate metabolism (Supplementary Materials Table S2). Additional pathways were found to be statistically relevant using mummichog pathway (https://shuzhao-li.github.io/mummichog.org/, accessed on 3 March 2021) assessment including TCA cycle, glyoxylate metabolism or valine, leucine, isoleucine biosynthesis pathways (Supplementary Materials Table S3). To illustrate these changes, some statistically significant exemplar metabolites were extracted from the matrix and presented as box and whisker plots (Figure 2C). The three TCA metabolites showed increases in the accessions from regions 1a and 1c which encompass the central Brachypodium subpopulation (and Bd21) compared to regions 2, 3 and 4, where the coastal subpopulation is located. Whilst most of the significant metabolite differences were seen as increases in the central subpopulation (Figure 1B), auxin, indoleacetic acid (IAA) and rhamnose were elevated in the coastal subpopulation.

### 3.2. Drought Stress and Metabolome Analysis

**Figure 3:**
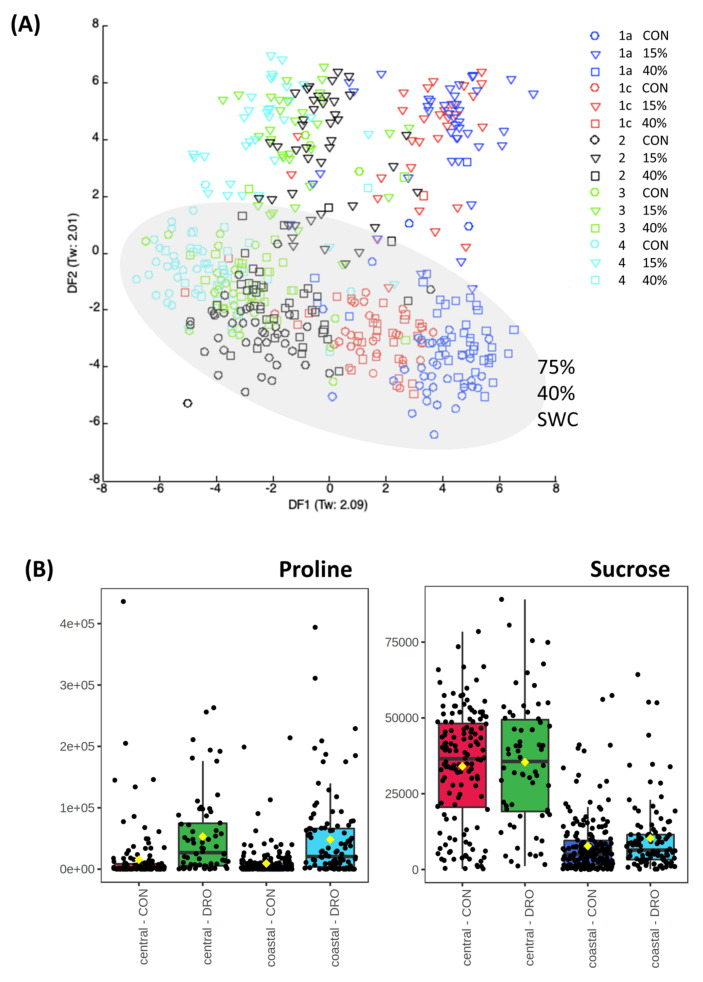
The metabolomes of Brachypodium accessions under drought stress and in controls. Eight-week-old Brachypodium accessions were transferred to the National Plant Phenomics Centre, Aberystwyth, UK. The watering was regulated to achieve three different soil water contents (SWC); 15%, 40% and 75% (control, CON). After 12 days of drought, leaves were sampled and the metabolomes were assessed using FIE-HRMS. (**A**) Discriminant Function Analysis of the derived metabolite profiles. Accessions are classified according of Turkish climate region of origin [6]: 1a (blue symbols), 1c (red symbols), 2 (black symbols), 3 (green symbols) and 4 (turquoise symbols). The 75% and 50% symbols are highlighted with a grey ellipsis to highlight their similarity but has no mathematical relevance. (**B**) Metabolomes from control (40%, 75% SWC) and droughted (15% SWC Brachypodium accessions forming the central (region 1a, 1c) and coastal (region 2, 3, 4) subpopulations were assessed for variation. Functionally important metabolites in drought responses; proline and sucrose are shown.

**Figure 4:**
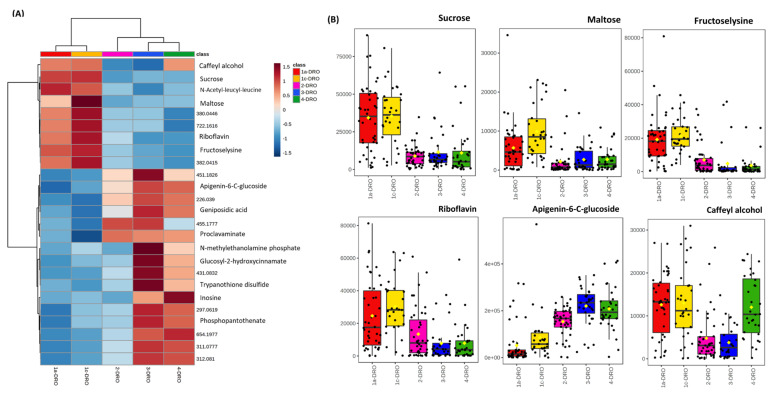
Key variables in the metabolomes of droughted Brachypodium accessions collected from different Turkish regions. Eight-week-old Brachypodium accessions were transferred to the National Plant Phenomics Centre, Aberystwyth, UK. The watering was regulated to achieve three different soil water contents (SWC): 15%, (droughted, DRO) 40% and 75% (control, CON). After 12 days of drought, leaves were sampled and the metabolomes were assessed using FIE-HRMS. The key variables discriminating between the droughted samples were identified by one way ANOVA (corrected for false discovery rates, FDR). (**A**) The relative levels of the *m/z* are displayed using a heatmap. Some mass-ions (m/z) were identified from comparison with databases and are labelled with names. Where *m/z* could not be identified these are given as the raw data values. (**B**) Box and whisker plot of exemplar metabolites which are increased in the central subpopulation (Turkish climatic region 1a, 1c) [6].

Metabolotypic differences between the regions and populations could also be revealed following the imposition of stress. As we have previously suggested that drought is a probable environmental driver for the variation between the two Brachypodium subpopulations, therefore the metabolomic responses to this stress were determined. Brachypodium accessions were grown under environmentally controlled conditions and watered to achieve three different SWC; 15%, 40% and 75%. Data describing the different growth of the accessions under drought conditions have been presented in [6]. After 12 days of drought, leaves were sampled and the metabolomes were assessed using FIE-HRMS (Figure 3A). 40% and 75% SWC data were similar and therefore, in further analyses, were considered together as controls. However, this control group exhibited subpopulation specific variation as noted in Figure 1. The sources of variation within each subpopulation were defined by ANOVA (*p* < 0.05). Amongst the metabolites that we targeted, we noted that proline levels increased with drought in both subpopulations (Figure 3B). As proline is an established indicator of stress this implied that both subpopulations were responding to the drought stress. Interestingly, we also noted that sucrose was significantly elevated in the central compared to the coastal subpopulation. However, this did not change in response to stress but could still represent a constitutive source of protection against drought based on increased osmolarity. This constitutive difference led us to explore metabolites which differed in the droughted samples from accessions originating from different Turkish regions (Figure 4A). Although these did not show a significant difference from control samples, metabolites were noted that could be functional in drought tolerance. In particular, we noticed higher concentrations of maltose and caffeoyl alcohol (Figure 4B) in the central subpopulation.

**Figure 5:**
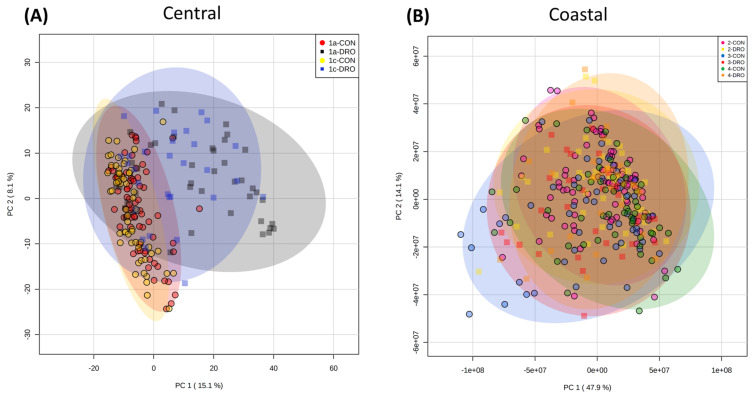
The metabolomes of Brachypodium central and coastal accessions under drought stress and in controls. Eight- week-old Brachypodium accessions were transferred to the National Plant Phenomics Centre, Aberystwyth, UK. The watering was regulated to soil water contents (SWC) of 15% (droughted, DRO), 40% and 75% (controls, CON). After 12 days of drought, leaves were sampled and the metabolomes were assessed using FIE-HRMS. Principal component analysis of Brachypodium accessions within the (**A**) central (Turkish climatic regions 1a, 1c) and (**B**) coastal (Turkish climatic regions 2, 3, 4) subpopulations [6].

**Figure 6:**
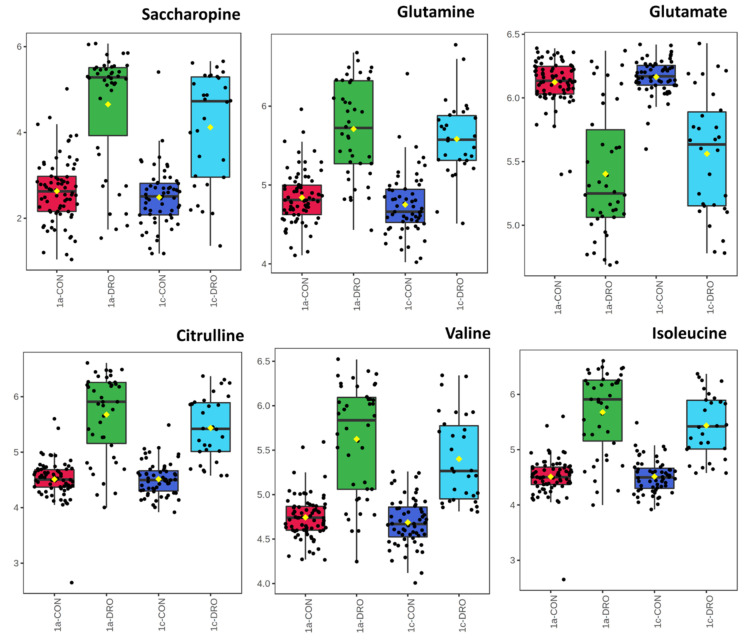
Amino acid changes seen in the central subpopulations in response to drought. Eight-week-old Brachypodium accessions were transferred to the National Plant Phenomics Centre, Aberystwyth, UK. The watering was regulated to soil water contents (SWC) of 15% (droughted, DRO), 40% and 75% (controls, CON). After 12 days of drought, leaves were sampled and the metabolomes were assessed using FIE-HRMS. Key variables changing in Brachypodium accessions within the central subpopulation (Turkish climatic regions 1a, 1c) [6] with drought and related to amino acid are displayed using box and whisker plots.

**Figure 7:**
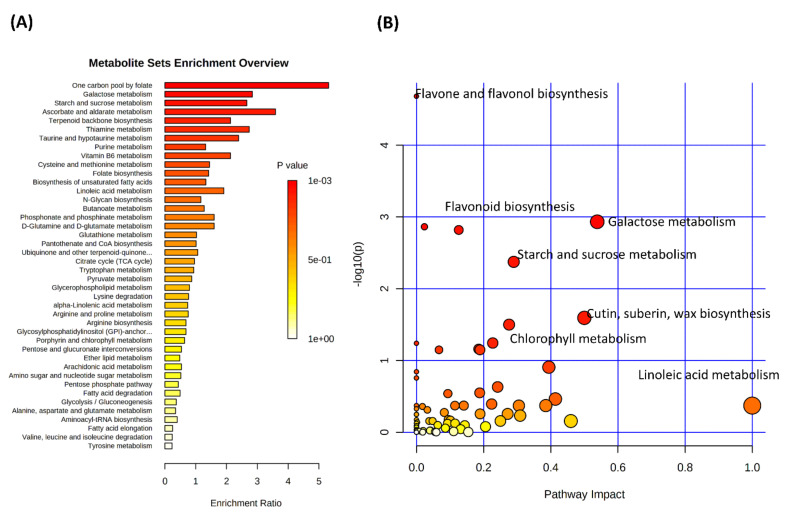
Enrichment analysis of the key metabolites responding to drought on the Brachypodium central subpopulation. Eight-week-old Brachypodium accessions were transferred to the National Plant Phenomics Centre, Aberystwyth, UK. The watering was regulated to soil water contents (SWC) of 15% (droughted, DRO), 40% and 75% (controls, CON). After 12 days of drought, leaves were sampled and the metabolomes were assessed using FIE-HRMS. Key variables changing in Brachypodium accessions within the central subpopulation (Turkish climatic regions 1a, 1c) [6] with drought and related to amino acid were identified and assessed using (**A**) metabolite set enrichment analysis (MSEA) or (**B**) the mummichog algorithm where significantly enriched pathways are also ranged for biological impact.

To reduce the influence of background genotypic metabolomic differences we considered variation in central and coastal subpopulations separately (Figure 5A,B). PCA of the central subpopulation showed that many accessions showed a response to drought (Figure 5A). In contrast, no clear response to drought was seen in the coastal accessions (Figure 5B). The source of variation in the central drought response were identified and ranked according to *p* value (Figure 6). This suggested that when considering individual metabolite changes there were changes in a range of amino acids (glutamate, glutamine, citrulline, leucine and valine) and saccharopine. To consider more wide-ranging differences, pathway enrichment (Figure 7A) and pathway impact analyses (Figure 7B) were undertaken based on the significant metabolite. These suggested statistically significant (Holm *p* < 0.05, FDR < 0.05) changes in galactose metabolism, pentose phosphate pathway and glycolysis/gluconeogenesis (Supplementary Materials Table S4). Therefore, changes in sugar and bioenergetic metabolism were prominent in the responses for the central subpopulation to drought.

## 4. Discussion

Metabolotyping is an emerging approach whereby individuals within a population or populations can be classified based on their metabolite profiles. The virtue of this approach, as opposed to assessments of genetic diversity, is that it can more immediately reveal functional changes. Indeed, metabotyping based on metabolomic approaches has been suggested to reflect the summation of genetic, transcriptional and post translational effects [15,37]. The metabotyping concept was first developed in a clinical context, particularly for defining diet and population-based studies of risks of cardiac disease [38,39,40]. Although not designated as “metabolotyping”, studies of plant metabolite diversity in populations are well established. Thus, metabolite variation in plants has been linked to geographical and environmental differences [41,42,43,44,45]. Often these assessments of metabolite variation are compared to genetic differences and a logical consequence of this is the development of metabolite genome-wide association study (mGWAS) approaches [46]. In the case of Brachypodium, there have been no wide-ranging studies of metabolite diversity, with the nearest being a comparison on the metabolomes of *B. distachyon*, *B. stacei* and *B. hybridum* [26]. We have recently established a new collection of Brachypodium accessions which were sampled across different climatic regions of Turkey. Following genomic sequencing and assessment of SNP variation, the accessions could be discriminated between costal and central subpopulations. Crucially, drought and possibly flowering time were suggested as selection pressures that might have led to differentiation between the subpopulations. Additionally, allopatric effects linked to the Anatolian plateau could have been factor [6]. Currently the number of accessions (52) that have been sequenced are insufficient to attempt mGWAS approaches, but we assessed how metabolomes vary in our Turkish collection.

Initially, we concentrated on unstressed seedlings that had been grown under identical non-stressful environmental conditions. Metabolite profiles were derived using FIE-MS in order to capture as much of the metabolomic variation as possible. When considered at one the basis of Turkish region of origin, considerable variation in the whole metabolomes was seen. However, separation between regions 1a and 3 and 4 was observed, although regions 1c and 2 showed some overlap (Figure 1). These groupings broadly corresponded to the regions from which the central and coastal subpopulations were defined. This became clearer when the most significant sources of variation were identified and these more clearly distinguished between the two subpopulations. Therefore, genetic variation appeared to be mirror with metabolomic and we here tentatively suggest could present two Brachypodium metabolotypes in Turkey.

To link these metabolotypes to function, metabolites that differed between the subpopulations were identified. These were associated with bioenergetic, sugar and amino acid metabolism. Plotting some of the most prominent changes indicated increases in the pyruvate and TCA metabolites in the central subpopulation (Figure 2). Such bioenergetic changes are well- characterised in responses to various stresses [47,48,49] and these have been linked to increased carbon flux through the TCA cycle to meet the bioenergetic demands of stress tolerance [50]. However, in this case, we observed these difference in seedlings that were, as far we are aware, unstressed. The lack of a significant difference in the levels of proline (Figure 3B), a marker of stress [51], in non-droughted Brachypodium accessions would support this hypothesis. As a result, it is likely that the central subpopulation is metabolomically pre-programmed to be bioenergetically primed to be stress tolerant.

Comparing the coastal and central subpopulations, we saw a potentially important difference in the elevated levels of IAA in the former (Figure 2). Auxins are well known growth regulators with wide roles [52]. This is a particularly interesting finding in that we found no significant differences between the coastal and central subpopulations when we assessed above ground plant growth, at least at seedling stage [6]. IAA is both a short and a long-range signalling molecule, so IAA made in the leaves could affect root behaviour. For example, Reed et al. 1998 [53] showed, for *Arabidopsis thaliana* (Arabidopsis), that inhibition of IAA transport from the shoot impacted lateral root formation. In roots, growth rates are inversely proportional to the IAA concentration and this reflects shoot to root polar transport. Under drought conditions, root length can increase to better access soil water and thereby enhance tolerance [54]. The relationship between drought tolerant/sensitive phenotypes in the different subpopulations, their root architectures and the different IAA levels in shoots should be explored further.

Next, the impact of drought on the Brachypodium metabolomes was directly assessed. This suggested that the concept of drought tolerance/susceptibility in our Brachypodium accessions was relative, with all accessions displaying no discernible metabolomic changes when grown in 40% as compared as 75% SWC. Thus, as a species, Brachypodium displays a degree of drought tolerance as we have previously noted [31] and is consistent with its preferred habitats. Initial assessments of the metabolomes from the 15% SWC drought experiment suggested some constitutive metabolite differences, which could contribute to the relative differences in drought tolerance seen between the coastal and central subpopulations (Figure 4). Most tellingly, changes in osmolytically active metabolites (sucrose, maltose and fructoselysine) are significantly elevated in the central subpopulation compared to coastal to go with important changes in such as proline seen in both subpopulations. Therefore, the central population metabolotype would appear to have “built in” drought tolerance [55]. Fructoselysine (fructosamines are the products of a non-enzymatic reaction of glucose with primary amines) is an unusual metabolite that has not been extensively studied in plants. Precedents from assessments of bacterial metabolism suggest that fructosamines could contribute to bioenergy metabolism, as well as possible osmoregulatory roles [56]. The constitutive elevation in riboflavin (vitamin B2) in the central subpopulation is also likely to contribute to drought tolerance. As a vitamin it contributes co-factors which play important roles in normal plant growth and development. Moreover, within a drought context, riboflavin can increase the activities of superoxide dismutase (SOD), catalase (CAT), ascorbate peroxidase (APX) and glutathione reductase (GR) and thereby could reduce the impact of stress-linked reactive oxygen generation [57]. The clearest increases in the coastal subpopulation were with apigenin-6-C-glucoside (isovitexin). This flavone has anti-oxidant properties [58] and such polyphenolics could contribute to stress tolerance [59]. Such aspects could be important in the coastal subpopulation.

Whilst such constitutive differences could be important in the coastal subpopulation, drought (15% SWC) did not have any significant observable effect on their metabolomes (Figure 5B). This was not the case with the central subpopulation (Figure 5A) where drought has an effect and these seemed to be linked to amino acid changes (Figure 6). The elevation of glutamate and glutamine could be indicative of elevated nitrogen uptake during drought and this would be reflected in other amino acid levels as seen here and which included the lysine metabolite intermediate, saccharopine. Interestingly, the metabolism of saccharopine leads to the production of glutamate [60]. These observations suggest that glutamate is important for drought tolerance in Brachypodium. This would align with the growing appreciation of glutamate as a signal in its own right that contributes to tolerance against a range of biotic and abiotic stresses [61]. This topic has been revolutionised by the characterisation of glutamate receptors (GLR) in plants, and a series of GLR mutants, primarily in Arabidopsis, have demonstrated the importance of glutamate in salt, heat and drought stress [61]. With only a few examples of GLR stress studies in cereals [62], Brachypodium could be an ideal model to develop our understanding of glutamine signalling in grasses.

Pathway enhancement analyses suggested that there were changes in sugar/starch, anti-oxidant (ascorbate) production and polyphenolic (flavonoid, flavone and flavonol) metabolism (Figure 7). These appeared to be standard biochemical responses to the drought associated the provision of bioenergetic resources and responses to oxidative stress and osmotic shock. These were all prominent in the central subpopulation.

Taking our findings together, the genetic and methylomic variation that led us to define subpopulations (central and coastal) are strongly reflected as distinct metabolotypes within the Turkish Brachypodium accessions. Furthermore, characterisation of the differences between the subpopulations reveals metabolite profiles which could provide a selective advantage for stress tolerance, particularly in the central subpopulation. Thus, the prevailing or past conditions in the different climatic zone seem to have left a metabolic signature that may represent adaptation to water stress. Given the ongoing refinement of metabolomic technologies, assessment of metabolite changes reflecting stress and species diversity in Arabidopsis [63], wheat [64] and *Pilocarpus pennatifolius* [65] promises to provide new insights into the evolution of plants and the adaptations associated with different climatic conditions. Given predictions of rapid climate change, there is an urgent need to quickly assess our crops for their ability to tolerate stress. The undomesticated model grass, Brachypodium, is a useful species in which to develop and test these approaches.

## Data Availability

The data presented in this study are available as supplementary files.

## Supplementary Materials

The following are available online at https://www.mdpi.com/2073-4409/10/3/683/s1. Table S1: Metabolite profile matrices of Brachypodium accessions. Table S2: Significance levels for each pathway for Brachypodium T0 generation. Table S3: Significance levels for each mummichong pathway assessment for Brachypodium T0 generation. Table S4: Significance levels for each pathway for Brachypodium central subpopulation after drought stress.
